# Measuring mental health burden in humanitarian settings: a critical review of assessment tools

**DOI:** 10.1080/16549716.2020.1783957

**Published:** 2020-07-13

**Authors:** Ashley Moore, Joris Adriaan Frank van Loenhout, Maria Moitinho de Almeida, Pierre Smith, Debarati Guha-Sapir

**Affiliations:** a Department of Social and Behavioral Sciences, Yale University School of Public Health, New Haven, CT, USA; b Centre for Research on the Epidemiology of Disasters (CRED), Institute of Health and Society, Université Catholique de Louvain, Brussels, Belgium; c Institute of Health and Society IRSS, Université Catholique de Louvain, Brussels, Belgium

**Keywords:** Disasters, mental health assessment, emergencies, humanitarian, trauma

## Abstract

**Background:**

The effects of disasters and conflicts are widespread and heavily studied. While attention to disasters’ impacts on mental health is growing, mental health effects are not well understood due to inconsistencies in measurement.

**Objective:**

The purpose of this study is to review mental health assessment tools and their use in populations affected by disasters and conflicts.

**Method:**

Tools that assess posttraumatic stress disorder, depression, substance use disorder, and general mental health were examined. This review began with a search for assessment tools in PubMed, PsycINFO, and Google Scholar. Next, validation studies for the tools were obtained through snowball sampling. A final search was conducted for scientific studies using the selected tools in humanitarian settings to collect the data for analysis. The benefits and limitations described for each tool were compiled into a complete table.

**Results:**

Twelve assessment tools were included, with 88 studies using them. The primary findings indicate that half of the studies used the Impact of Events Scale-Revised. The most common limitation discussed is that self-report tools inaccurately estimate the prevalence of mental health problems. This inaccuracy is further exacerbated by a lack of cultural appropriateness of the tools, as many are developed for Western contexts.

**Conclusion:**

It is recommended that researchers and humanitarian workers reflect on the effectiveness of the mental health assessment tool they use to accurately represent the populations under study in emergency settings. In addition, mental health assessment should be coupled with action.

## Background

Disasters and conflicts create humanitarian crises that occur globally and affect millions of people yearly. A humanitarian setting is a setting in which a natural or manmade disaster or civil conflict occurs that exceeds local coping capacity and requires external assistance or humanitarian action [1]. In 2018, 315 natural and technological disasters occurred [2]. The majority are natural, and most disasters from 1998 to 2017 were extreme weather events, such as floods, droughts, and heat waves [3]. Other natural and technological disasters include earthquakes, hurricanes, and large-scale accidents. Interest in their mental health effects has grown due to the potential for trauma. Synthesized research about disaster mental health shows that posttraumatic stress disorder (PTSD), major depressive disorder, and substance use disorder are common outcomes [4]. Other outcomes of interest include generalized anxiety disorder (GAD), prolonged grief, panic disorders, and phobias; however, these outcomes are less frequently studied than PTSD, depression, and substance use [4]. In addition to natural and technological disasters, conflicts and related displacement greatly contribute to the global population in need of humanitarian assistance. Mental health research in humanitarian settings is heavily focused on PTSD and indicates that the prevalence of PTSD and depression in these settings is much higher than in the general population [5].

Though the morbidity and mortality of conflict-affected populations are decreasing due to effective disease control programs, these populations continue to face safety concerns with the prolonged nature of contemporary conflicts [6]. Furthermore, conflict research shows that civilians who experience war conflicts, especially women and children, are at a high risk for persisting mental health effects [7]. Displacement contributes to stress and is associated with loss of a loved one, destruction of the home, and limited access to stable resources [7]. The damage to infrastructure that conflicts bring to communities removes access to mental health resources and exacerbates individuals’ stress [7].

Great variability exists among the methods of evaluating mental health in humanitarian settings [4]. The lack of standardization in assessment approaches hinders researchers’ and humanitarian organizations’ ability to ascertain the true impact of disasters on mental health. For example, a systematic review of literature up to November 2013 on the mental health outcomes of Iraqi refugees in Western countries shows the prevalence of PTSD and depression ranging from 8% to 37% and 28% to 75%, respectively [8]. In-depth diagnostic interviews may be the gold standard for such measures, but research in humanitarian settings warrants more brief and easy-to-use tools that measure only symptoms and thus do not require the presence of a clinician. In addition, rapid screening tools can be useful in decision-making and program planning due to their ability to obtain the burden of mental distress in a time-limited setting. The purpose of this critical review is to evaluate the use of different tools for studying or assessing the mental health effects of disasters and conflicts. The outcomes of interest are PTSD, depression, anxiety, substance use disorder, and general mental health and were chosen due to their high prevalence in disaster and conflict research.

## Methods

Three searches were conducted for this review: the first search collected commonly used mental health assessment tools, the second collected their validation studies, and the third collected studies that used these tools in disaster or conflict mental health research.

### Assessment tool search

A list of mental health assessment tools was compiled using Google Scholar, PsycINFO, and PubMed search engines. Each tool had to be individual, brief, developed in or after 1990, and non-diagnostic to be included in the study. A combination of the following MeSH keywords was used for this search: ‘symptom assessment,’ ‘standards,’ ‘emergencies,’ ‘disasters,’ ‘humanitarian assistance,’ ‘mental health,’ ‘posttraumatic stress disorder,’ ‘depression,’ ‘substance use disorders.’ We employed snowball sampling to obtain comprehensive information about the tools and ascertain which tools are commonly used, since we had limited initial information regarding the properties of commonly used tools in emergency settings. The length, purpose, and existence of translations for each tool were ascertained. We excluded tools that evaluate community needs, assess lifetime mental illness, or involve in-depth interviews. We selected the most recent version if multiple versions of the tool existed.

### Validation study search

We then conducted a search on PubMed and Google Scholar and obtained psychometric properties and validation studies to present consistency in validation and the presence of cross-cultural applications of the tools in the existing literature, regardless of population or setting. Validation studies include studies in which researchers determine if the tool adequately distinguishes between a distressed and a non-distressed person, and the tools are often validated against an existing widely used tool such as the General Health Questionnaire. For this search, we did not employ MeSH search terms; rather, we searched the terms ‘[assessment tool]’ and ‘validation study’ and recorded the studies that affirm or deny the validity of the tool in specified languages and/or populations.

### Study search

Finally, we conducted a targeted review of peer-reviewed literature that has used one of the selected assessment tools in humanitarian settings, using both PubMed and Google Scholar. For this final search, a combination of the following MeSH keywords was employed: [assessment tool (not MeSH)] *and* ‘natural disasters,’ ‘armed conflicts.’ An experienced librarian at UCLouvain validated the search methodology. Inclusion and exclusion criteria are summarized in Table 1.

**Table 1.** Inclusion and exclusion criteria for studies on MH assessment tools^a.^

Criteria type	Inclusion	Exclusion	Justification
Population	Civilians of any age affected by a disaster or conflict	Veterans	Veteran populations likely have vastly different experiences than civilian populations, which would fragment the findings.
Intervention/exposure of interest	Human-made intentional (conflict/war), human-made unintentional (technological), natural	Terrorism	The focus of this paper is on events that result in a humanitarian crisis, and thus excludes terrorism events.
Comparison	N/A	N/A	The review did not limit studies based on inclusion of comparison groups.
Outcome	Any mental health outcome	N/A	While there were no exclusion criteria for outcome of interest, the mental health outcomes naturally were limited to PTSD, depression, substance use, and general mental health due to the nature of mental health research in humanitarian settings.
Study type and year	Epidemiological studies conducted in 2000–2019	Systematic or literature review; intervention evaluation; studies conducted before 2000	Review and evaluation articles are excluded due to the likelihood of a repetition in citations collected. Studies published before 2000 were excluded to ensure timeliness and feasibility.

^a^Table follows PICO format, where applicable [9].

If no studies corresponded with a particular tool, then that tool was dropped from the list, as we could not provide an adequate recommendation without evidence of the tool’s utility.

Doubts regarding study or tool eligibility were discussed between AM, MMA, PS, and JvL.

We extracted the benefits and limitations cited in each study regarding the particular tool and its utility in populations affected by disaster or conflict. Based on these observations, we described the main strengths and weaknesses of each tool in assessing the mental health outcomes in these populations.

## Results

### Assessment tool search results

**Figure 1.**
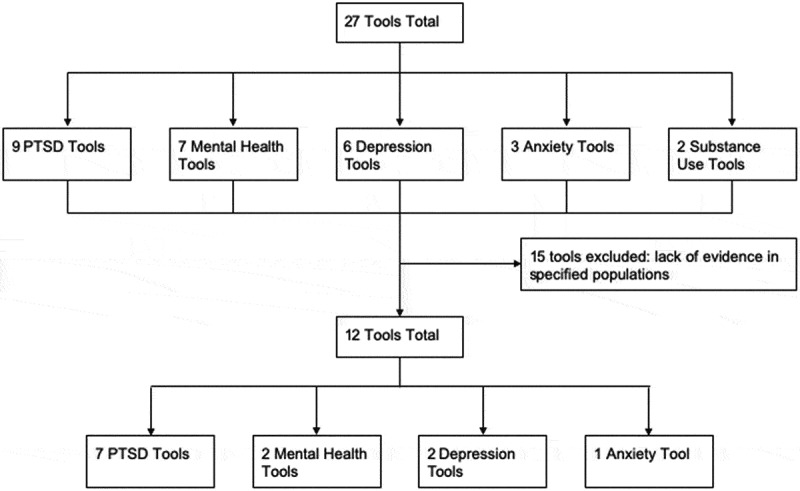
Flowchart for selection of mental health assessment tools in disaster- and conflict-affected populations resulting in 12 tools.

The assessment tool search resulted in a total of 27 tools for analysis consisting of nine tools for PTSD, seven tools for general mental health, six tools for depression, three tools for anxiety, and two tools for substance use disorder (Figure 1). Fifteen tools were excluded from the study due to a lack of evidence regarding their use in populations affected by disaster or conflict. Twelve tools remained for analysis: seven tools for PTSD, two tools for general mental health, two tools for depression, and one tool for anxiety. We did not identify any tools that evaluated substance use disorder that matched our eligibility criteria. Three tools, the Posttraumatic Symptom Scale – Self Report, SPAN, and Davidson Trauma Scale, required payment to view the full tool details but were nevertheless included due to adequate secondary information.

Table 2 presents the year published, psychometric properties, and symptom period of the tools. Most tools exhibit high reliability and validity for the populations in which they were originally developed. Tool length ranges from 4 to 33 items and takes between 5 and 10 minutes. The tools also specify that symptoms should last between 1 week and 1 month.

**Table 2.** Psychometric properties.

Tool	Year	Validity	Reliability	Length	Symptom period, time
**Anxiety tool**					
Beck Anxiety Inventory (BAI) [10]	1993	Good discriminant validity	Alpha = 0.92	21 items	1 month 5–10 minutes
**Depression tools**					
Beck Depression Inventory II (BDI-II) [11]	1996	Good content and convergent validity	Alpha = 0.93	21 items	2 weeks 5 minutes
Patient Health Questionnaire 9 (PHQ-9) [12]	1999	Good criterion, construct, and external validity	Alpha = 0.89	9–10 items	2 weeks Unknown time
**PTSD tools**					
Children’s PTSD Symptom Scale (CPSS) [13]	2001	Convergent validity = 0.80; 95% of cases were correctly identified	Alpha = 0.89	24 items	2 weeks 10 minutes
Davidson Trauma Scale (DTS) [14]	1997	Good concurrent, construct, and predictive validity	Good test-retest and split-half reliability and internal consistency	17 items	1 week 10 minutes
Impact of Events Scale – Revised (IES-R) [15]	1997	Construct validity = 0.84	Alpha = 0.96	22 items	7 days Unknown time
PTSD Checklist – Specific (PCL-S) [16]	1993	Good convergent validity	Good test-retest reliability and internal consistency	20 items	1 month 5–10 minutes
PTSD Symptom Scale – Self Report (PSS-SR) [17]	1993	Concurrent validity = 0.68	Good test-retest reliability and internal consistency	17 items	Unknown
Posttraumatic Cognitions Inventory (PTCI) [18]	1999	Good convergent and discriminant validity	Alpha = 0.97	33 items	Unknown
SPAN Self-Report Screen (SPAN) [19]	2002	Unknown	Unknown	4 items	1 week Unknown time
**General mental health tools**					
Screening Questionnaire for Disaster Mental Health (SQD) [20]	2007	Convergent validity = 0.94	Alpha = 0.83	12 items	1 week Unknown time
WHO-UNHCR Assessment Schedule of Serious Symptoms in Humanitarian Settings (WASSS) [21]	2012	Unknown	Unknown	6 items plus a household roster	2 weeks 7–8 minutes

### Validation study search results

Table 3 presents the validated populations and languages for each tool. The tools have been validated across a variety of different populations and regions. The PHQ-9 had the most validation studies backing it. Most of the tools have been validated in a language other than English. The PSS-SR and the WASSS are the only tools with no validation studies.

**Table 3.** Validation studies.

Tool	Validated populations or methods	Validated languages other than English
**Anxiety tool**		
BAI	German patients [22] Chinese doctors [23] Psychiatric inpatient and high school adolescents [24]	German [22] Chinese [23] Portuguese [25]
**Depression tools**		
BDI II	Adolescent and adult inpatients [26,27,29] Low-income African American medical outpatients [30] American and Jamaican HIV*-positive patients [31,32] Family caregivers of children with chronic disease [33]	Portuguese [34] Croatian [35] Japanese [36] Korean [37] Xhosa [28]
PHQ-9	Patients with epilepsy, migraine, multiple sclerosis, stroke, spinal cord injury, traumatic brain injury, Parkinson’s disease [38–43] Chilean adolescents [44] Primary care in South Africa [45] Iranian psychiatric outpatients [46] Korean American elderly [47] Nepal, with added idioms of distress [48] MSM* in Haiti [49] Germans and Turkish immigrants in Germany [50] Pregnant women [51] Polish hospitalized elderly [52] Administered through interactive voice technology [53] Patients with Type 2 Diabetes in Malawi and The Netherlands [54,55] Employees on sick leave [56] Pregnant women in Ethiopia [57] Chinese Americans in primary care [58] General population in Hong Kong [59]	Chinese [60] Korean [61] Malayalam [62] Portuguese [63] Polish [52] Latvian and Russian [64] Afaan Oromo [57] Japanese [65]
**PTSD tools**		
CPSS	N/A	Hebrew [66] Spanish [67] Nepali [68]
DTS	Chilean people exposed to F-27 earthquake [69] Military veterans served after 9/11 [70]	Korean [71] Chinese [72] Spanish [73]
IES-R	Women exposed to disaster before or during pregnancy [74] Adolescents exposed to typhoon in Taiwan [75] Swedish patients with burns [76]	French [74] Chinese [77] Japanese [78] Malay [79] Tamil [80]
PCL-S	Norwegian survivors of 2004 Southeast Asian tsunami [81]	Japanese [82]
PSS-SR	N/A	N/A
PTCI	Brazilian population [83]	N/A
SPAN	N/A	Chinese [84] Korean [71]
**General mental health tools**		
SQD	People affected by earthquake in Japan [20]	Italian [85]
WASSS	N/A	N/A

*HIV: Human Immunodeficiency virus; MSM: men who have sex with men.

### Study search results

**Figure 2.**
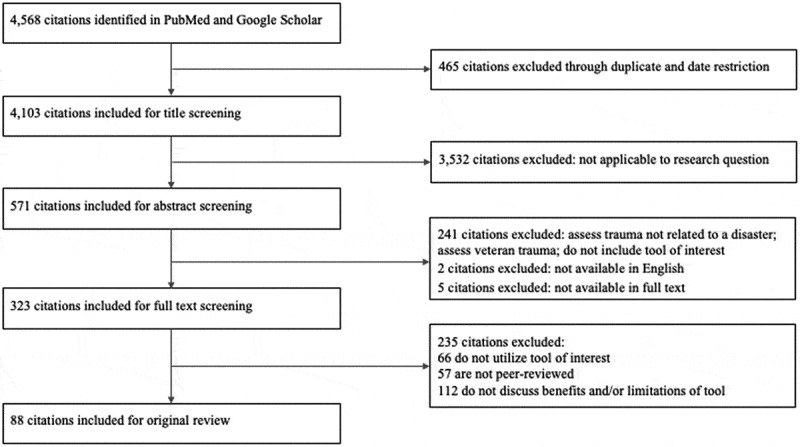
Flowchart of article selection for studies that used an assessment tool from previous search resulting in 88 total citations.

Of the 86 studies included in the review (Figure 2), 82 focused on people affected by natural and technological disasters and four focused on people affected by conflict. Thirty-four different disasters were studied. The 2008 Wenchuan earthquake and 2005 Hurricane Katrina were the top two most frequently studied disasters with 17 and nine studies, respectively. Of the four studies that examined the effects of conflict, three focused on people affected by the Georgian conflict and one focused on those living in the Gaza strip. All tools but the SQD originated in English. The SQD originated in Japanese but was translated into English for validation. The greatest number of tools was available in Nepali, while the greatest number of studies used a Chinese translation of the tools. Other translations may be available for the selected tools but were not identified due to lack of validation.

The main strengths and limitations for each tool are presented in Table 4. The IES-R, measuring PTSD symptoms, is by far the most widely used tool among all of the studies, with 44 of the 86 studies using it. The second most widely used tool among the studies is the CPSS, with 11 studies using it to study the posttraumatic effects of crises on children.

**Table 4.** Tool strengths and limitations.

Tool, # of studies*	Strengths	Limitations	Populations studied and languages other than English used
**Anxiety tool**			
BAI 5 studies	Availability of multiple languages [87]	Not culture-specific [87] Tool is in English, not Tibetan language [91] May not be extrapolated to other populations [92] Not validated in Haitian contexts and Western tools may not be appropriate [93] Self-report [94]	● Tibetan refugees in North India [91] ● Chinese elderly 2013 Ya’an earthquake survivors [92] ● 2010 earthquake-exposed Haitians in Florida [87,93] ● 2008 Iceland earthquake survivors [94] Language(s): ● Icelandic [94]
**Depression tools**			
BDI II 4 studies	Can be administered online, convenient, accessible [95] Widely used [96]	Self-report and subjective [94–97] Valid Nepali language version does not exist [97]	● 2008 Iceland earthquake survivors [94] ● Pet owners who survived Hurricane Katrina [95] ● Parents of internally displaced children in Georgia [96] ● Nepalese 2015 earthquake survivors in Phulpingdanda village [97] Language(s): ● Icelandic [94] ● Georgian [96] ● Nepali [97]
PHQ-9 9 studies	Adequate clinical applications [98] Diagnostically accurate estimate of prevalence [99]	May not be culturally sensitive to Georgian population [88,89] Self-report measure, not diagnostic [98–103] Conducting survey in-person for illiterate participants may skew results [104]	● Adults affected by conflicts in Georgia [88,89] ● Women displaced by Hurricane Katrina [98] ● Galveston Bay survivors of Hurricane Ike [100] ● Survivors of the 2016 Fort McMurray, Canada wildfire [99] ● 2009 Australia bushfire disaster [101] ● Workers who experienced the Great East Japan Earthquake [102,103] ● Survivors with spinal cord injury from the 2015 Nepal earthquake [104] Language(s): ● Georgian [88,89] ● Nepali [104]
**PTSD tools**			
CPSS 11 studies	Self-report measures can be valuable [86] Not a significant time burden [105]	Validated measures may produce significant results [86] Self-report measure, not diagnostic, overestimate [105–113] Between- and within-population variability in scores [114]	● Children who experienced 2010 Nashville, Tennessee flood [86] ● School children who survived Hurricane Katrina [105] ● Children and students in Phulpingdanda village who experienced 2015 Nepal earthquakes [106,114] ● Adolescent survivors of the Wenchuan earthquake [107,108,110–113] ● Children who experienced 2013 Ya’an earthquake [109] Language(s): ● Chinese [107–112] ● Nepali [114]
DTS 3 studies	Early detection, useful screening tool [115] Available in several languages [116]	Designed for screening, may not catch people with acute PTSD [117]	● Survivors of 2005 Pakistan earthquake [115] ● Residents during 2017 earthquakes in Mexico [116] ● Rescue workers in 1999 Chi-Chi earthquake [117] Language(s): ● Urdu [115]
IES-R 44 studies	Helpful for initiating treatment programs [118] Symptom assessment and comparison of a large number of people [119] Useful in time-limited situations [120] Useful for in-person surveys with low literacy populations [121]	Not be totally reliable, may overestimate prevalence [118,119,122–151,173,174] Not formally validated in Nepalese population [138] Relies on DSM-IV criteria [152] No validity or reliability for Turkish, French, Tamil, Sinhalese version [153–155] Might not be culturally sensitive [156] Low-range scores may be misdiagnosed [157] May underestimate prevalence [158] Lack of a cutoff recommendation [150,159,173]	● Flood-affected adults in Tamil Nadu [118] ● Survivors of the 2013 North India floods [121] ● Swedish survivors of the 1994 MS Estonia disaster [122] ● People affected by Hurricane Sandy [123] ● Adult survivors, pregnant survivors of Wenchuan earthquake [124,125,133,142] ● General population, students, low-income parents who survived Hurricane Katrina [126,128,130,141,143] ● Survivors of mudslide and Wenchuan earthquake [127] ● Rescue workers of Great East Japan Earthquake [129] ● Survivors of 2000 Miyake Island volcanic eruption [131] ● Survivors of 2010 Canterbury, New Zealand earthquakes [137] ● Those who experienced 2014 flood in Malaysia [140] ● Treatment-seeking individuals who experienced the 2009 L’Aquila earthquake [144] ● Nuclear plant workers, evacuees who experienced the 2011 Fukushima disaster [145]
			● Chinese students who experienced 2008 snowstorm disaster [148] ● Survivors of 2012 Yiliang earthquakes [149] ● Israeli backpackers & mothers who experienced the 2015 Nepal earthquake [138,150] ● Adolescents and young adults who experienced the 2010 Haiti earthquake [152] ● Rescue workers in 1999 Marmara, Turkey earthquake [153] ● Survivors of 2001 factory explosion in Toulouse, France [154] ● Swedish, Norwegian tourists, Sri Lankan survivors who experienced the 2004 Southeast Asia tsunami [119,120,135,136,155] ● Responders to the 2005 Northern Pakistan earthquake [156] ● Adults, psychiatric patients, cardiovascular patients, Japanese adolescents, junior high students, and Qiang women who survived the Great East Japan Earthquake [132,134,139,146,157,173,174] ● Joso City residents who experienced 2015 Tokyo flooding [158] ● Tamil Nadu, India survivors of 2004 Southeast Asian tsunami [151] ● Survivors of the 2010 Mount Merapi volcano eruption [159] Language(s): ● Tamil [118] ● Swedish [119] ● Japanese [129] ● Nepali [138] ● Hebrew [150] ● Turkish [153] ● French [154] ● Sinhalese [155] ● Urdu [156] ● Bahasa Indonesian [159]
PCL-S 7 studies	Can compare results with other studies [102] Allowed for the collection of comprehensive data [160]	Self-report measure [102,103,161–163] May only assess acute stress symptoms if administered soon after a disaster [164] Not validated in China [165]	● Workers who experienced the Great East Japan Earthquake [102,103] ● Hypertensive adults who experienced Hurricane Katrina [160] ● Adult survivors of the 2014 flood disaster in Kashmir [161] ● Survivors of the Wenchuan and Lushan earthquakes [162] ● Survivors of Super Typhoon Haiyan [163] ● Adults who experienced Hurricane Harvey [164] ● Mothers who lost a child in the 2008 Sichuan earthquake [165] Language(s): ● Japanese [102] ● Chinese [162,165]
PSS-SR 2 studies	Able to be administered online, convenient, accessible [95]	Self-report, not appropriate for diagnostics [92,95]	● 2008 Iceland earthquake survivors [94] ● Pet owners who survived Hurricane Katrina [95] Language(s): ● Icelandic [94]
PTCI 1 study	None listed	Self-report measure, not objective [166]	● Palestinian mothers and their infants living in the Gaza strip [166] Language(s): ● Arabic [166]
SPAN 2 studies	None listed	Poorer diagnostic accuracy than DTS [117] Self-report may limit the strength of findings [167]	Rescue workers, survivors in 1999 Chi-Chi earthquake [117,167]
**General mental health tools**			
SQD 3 studies	Efficient and easy to use for time-limited situations [90] Can be used by those without expertise, self-reporting is easy [168]	Self-report tool [168,169] No formal validation in Indian population [168]	Adults exposed to the 2009 L’Aquila earthquake [90] Population affected by 2008 floods in Bihar [168] Older adults who experienced the Great East Japan Earthquake [169]
WASSS 1 study	Brief measure allows for the inference of mental health symptoms [170]	First use of the WASSS measure [170]	● Survivors of 2015 Nepal earthquakes [170] Language(s): ● Nepali [170]

*May not add up to 86 due to studies using multiple tools.

The most common strengths described for the screening tools are convenience and brevity. However, the limitations of the tools comprised the bulk of the information discussed in the studies. The most common limitation described for all tools, cited 64 times, is that a self-report screening tool is not diagnostic and can therefore over or underestimate the prevalence of the given disorder. However, some studies also list the self-report aspect as a benefit and state that it can provide valuable information about an individual’s wellbeing [86]. Another common limitation described is the lack of cultural sensitivity. Most of the tools were developed based on the Diagnostic and Statistical Manual (DSM) criteria, which were established by the American Psychological Association. The origins of many tools in this review may result in cultural bias, even if the tool has been validated in a certain population or translated to another language [87–89]. A lack of a suggested cutoff point for diagnosis is the third most common limitation among the studies. Some studies using tools such as the IES-R set their own cutoff point depending on the characteristics of the population and follow previous studies in similar settings. This provides versatility; however, it also lends to inconsistency. Comparisons across populations cannot be made if the cutoff is different for different studies.

The SQD and WASSS, though less frequently used than other tools, were designed particularly for humanitarian settings to briefly identify those in distress after a crisis. The SQD has been used more than the WASSS and is designed for time-limited situations [90].

## Discussion

This unprecedented review highlights the high number of existing mental health assessment tools that have been used in the context of disasters and conflict, as well as their benefits and drawbacks. We identified 12 assessment tools for further analysis, most of which have exhibited high reliability and validity in the populations for which they were originally developed. A systematic literature search uncovered 86 studies that assessed mental health in populations affected by disasters and conflict using one of these tools, half of which used the IES-R.

Differential use of assessment tools across studies contributes to the fragmentation of knowledge of the burden of mental health issues in humanitarian settings. Each tool has its own levels of sensitivity and specificity, especially those with variable cutoffs. Furthermore, the disorders have different latency periods from exposure to symptom manifestation, as accounted for by the symptom period specified in the tool characteristics. The timing of measurement can greatly affect estimated prevalence. This fragmentation not only impedes synthesis of knowledge of the effects of disasters and conflicts, but also might lead to multiple assessments of the same communities, resulting in increased emotional and time burden for them. In addition, the tools used may not be culturally appropriate for measuring mental health outcomes in these communities.

Most of the identified studies assessed PTSD symptoms. This was expected due to PTSD being the most studied outcome of disasters and conflicts, and the tool most used to study PTSD was the IES-R. The second most studied outcome was depression, for which most of the studies used the PHQ-9. Of all the tools, the PHQ-9 was the most frequently validated, indicating its wide usage outside of humanitarian research. Anxiety was the third most studied outcome and was measured by the BAI. General mental health, measured using the WASSS and SQD, was the least studied outcome.

While studies that measured the mental health effects of natural and technological disasters and conflicts were eligible for inclusion, the vast majority of studies in this review focused on natural disasters. Surprisingly, the only conflict-affected populations studied were those who lived in the Gaza strip and those who experienced the Georgian conflict, indicating a dearth in mental health research on civilians in conflict. Further, few studies measured the effects of technological disasters on population mental health, which may be due to a generally smaller impact size of technological disasters compared to natural disasters.

The primary limitation cited in the studies is that a self-report tool may result in inaccurate estimates of the prevalence of a disorder. Self-report screening tools are inherently not diagnostic, as they are designed to rapidly assess those with the highest likelihood of the outcome of interest. Using screening tools to measure the prevalence of a mental health outcome is problematic, because such tools were not designed to definitively assess an individual. However, the alternative ‘gold standard’ diagnostic interview is not feasible in humanitarian and emergency settings or for the purposes of medium-scale mental health projects without adequate funding. The benefit of screening tools for these purposes is that they are rapid, while diagnostic interviews are lengthy and require the presence of a clinician.

The cultural appropriateness of the tools is an important consideration when using the tools, especially in a global context. Cultural appropriateness of assessment methodology is one of the guiding principles of the Interagency Standing Committee’s (IASC) assessment of mental health in humanitarian emergencies [171]. Only one tool, the SQD, was developed in a non-western context. The tools in this review that were developed for high-income western populations and later translated and implemented in low- and middle-income countries could result in culturally insensitive questions, meanings lost in translation, and ultimately inadequate measurement of true effects. Because most assessment tools are based on DSM criteria, they are inherently western-based and may not produce valid findings in cross-cultural mental health research.

The third issue is the use of a cutoff in determining a diagnosis. A cutoff is set to balance between sensitivity and specificity, but not all items in a screening tool may be created equal yet are often weighted the same [172]. Individuals whose sum of symptoms breach the set cutoff could be at markedly different levels of distress than others due to the potentially varying importance of items in the questionnaire. In spite of this, cutoffs are sometimes necessary to estimate prevalence or quickly group those who need immediate assistance. If the purpose of the tool’s use is to build knowledge to develop programs for disaster mitigation, then inconsistent measures and cutoffs would hinder this goal [173,174]. Those who work in humanitarian response and research and use these tools must consult the evidence and experts to make an informed decision on where to set the cutoff.

Some tools have substantially more evidence of use, which might indicate that they are more suitable than others for mental health assessment. While abundant evidence allows for comparisons between and within populations in research, it does not necessarily mean that the tools accurately measure the prevalence of mental health outcomes. On the other hand, tools that were developed specifically for humanitarian situations may be more accurate than other tools when assessing the mental health of those affected by disasters and conflicts. However, these tools that specifically ask about a traumatic event cannot be used in a control group that has not experienced that event. In addition, tools such as the WASSS and the SQD are fairly new and thus do not allow for ready comparison between populations. The motivations behind the use of the assessment tools will ultimately determine which tool is most appropriate for a particular setting.

The importance of mental health assessment in crisis-affected populations is clear. Knowing these effects can inform preparedness and response to a large-scale trauma. However, individuals using these tools must consider the utility and implications of their use. As emphasized by the IASC, the needs of the crisis-affected populations should be prioritized.

### Strengths and limitations

The primary strength of this study is that it is among the first to analyze the benefits and limitations of a variety of tools that assess multiple mental health outcomes in populations affected by disasters. Much of the limited existing literature on this topic revolves around a single tool or mental health outcome or only discusses the psychometric properties of the tools [175,176]. In addition, the findings of this review can be used by both researchers and humanitarian workers since the tools included were designed for use in informal settings without the presence of a clinician. As the tools discussed are screening tools, they can be used to estimate prevalence and the care needs of the population to quickly identify those who are in distress.

Some limitations exist in this review. The search method for assessment tools was not systematic, and thus may have overlooked relevant tools or studies. However, the search was extensive and included a wide range of the literature. In addition, some tools may not have been identified through the snowball sampling method. However, this method allowed for a selection of a variety of tools with limited initial information and a reasonable number of tools have been included. Some tools require payment for access, and we were not able to fully examine them for analysis. Nonetheless, adequate information for these tools was available through secondary sources. Finally, the SQD and WASSS were recently developed, and there was little evidence of their use. This limited the conclusions that could be made about these tools. However, their inclusion in the review provided valuable information, as they were specifically designed for crisis-affected populations.

## Conclusion

The assessment of mental health in humanitarian settings is highly fragmented due to the use of a wide range of assessment tools. This review provided a thorough analysis on each of the identified tools. Moving forward, researchers and humanitarian workers must understand the implications of using brief mental health assessment tools in affected populations in order to better mitigate the impacts of future emergencies. This review provides the basis for further research on instruments to measure the mental health of populations affected by disasters and conflicts.

Three prominent gaps exist that must be addressed. First, there is no standard assessment tool for disaster and conflict settings. Second, little is known about assessment tool applicability to conflict settings. Third, these studies lack practical next steps to address the mental health outcomes they measure. Fortunately, greater awareness of mental health effects of mass trauma can motivate key stakeholders to close these gaps.

## Paper context

Disasters and conflicts exacerbate and induce psychological symptoms. However, the estimated prevalence of these conditions can vary depending on assessment tool. Little is known about which tools are most effective in measuring mental health in disaster and conflict settings. This paper outlines commonly used tools and provides recommendations based on the tool characteristics discussed by the studies reviewed. Researchers should consider these characteristics and choose the most appropriate tool for the study population.
